# Two-way tuning of structural order in metallic glasses

**DOI:** 10.1038/s41467-019-14129-7

**Published:** 2020-01-16

**Authors:** Hongbo Lou, Zhidan Zeng, Fei Zhang, Songyi Chen, Peng Luo, Xiehang Chen, Yang Ren, Vitali B. Prakapenka, Clemens Prescher, Xiaobing Zuo, Tao Li, Jianguo Wen, Wei-Hua Wang, Hongwei Sheng, Qiaoshi Zeng

**Affiliations:** 1 Center for High Pressure Science and Technology Advanced Research, Pudong, Shanghai, 201203 People’s Republic of China; 2 State Key Laboratory for Advanced Metals and Materials, University of Science and Technology Beijing, Beijing, 100083 People’s Republic of China; 3 Institute of Physics, Chinese Academy of Sciences, Beijing, 100190 China; 4 X-ray Science Division, Advanced Photon Source, Argonne National Laboratory, 9700 South Cass Avenue, Argonne, IL 60439 USA; 5 Center for Advanced Radiation Sources, University of Chicago, Chicago, IL 60637 USA; 6 Center for Nanoscale Materials, Argonne National Laboratory, Argonne, IL 60439 USA; 7 Jiangsu Key Laboratory of Advanced Metallic Materials, School of Materials Science and Engineering, Southeast University, Nanjing, 211189 People’s Republic of China

**Keywords:** Phase transitions and critical phenomena, Structure of solids and liquids, Glasses

## Abstract

Metallic glasses are expected to have quite tunable structures in their configuration space, without the strict constraints of a well-defined crystalline symmetry and large energy barriers separating different states in crystals. However, effectively modulating the structure of metallic glasses is rather difficult. Here, using complementary in situ synchrotron x-ray techniques, we reveal thermal-driven structural ordering in a Ce_65_Al_10_Co_25_ metallic glass, and a reverse disordering process via a pressure-induced rejuvenation between two states with distinct structural order characteristics. Studies on other metallic glass samples with different compositions also show similar phenomena. Our findings demonstrate the feasibility of two-way structural tuning states in terms of their dramatic ordering and disordering far beyond the nearest-neighbor shells with the combination of temperature and pressure, extending accessible states of metallic glasses to unexplored configuration spaces.

## Abstract (web summary)

While metallic glasses are expected to have tunable structures, these have rarely been demonstrated. Here, the authors combine temperature and pressure to show a two-way structural tuning in rare earth-based metallic glasses beyond the nearest-neighbor atomic shells.

## Introduction

Many crystalline materials can be readily altered between distinct structures via polymorphic phase transitions by controlling temperature and pressure. In contrast, metallic glasses (MGs)^1–3^ are disordered systems that lack identifiable structural symmetry. Consequently, MGs with different compositions seem to be almost indistinguishable in structures showing similar featureless patterns in high-resolution transmission electron microscope (HRTEM) images and also highly similar electron diffraction (ED) and x-ray diffraction (XRD) patterns^4^. On the other hand, MGs with the same compositions but different processing histories are often reported to have varied properties, implying that their seemingly alike atomic structures could be different and tunable to some extent.

The structural order in MGs can be described by the degree of deviation from a completely random atomic arrangement. After decades of studies, it has been recognized that MGs actually possess a short-range order (SRO) of the nearest-neighbor atoms^5,6^ and a medium-range order (MRO) on the length scales beyond the nearest neighbor^3,7,8^, or even a long-range topological order (LRTO) throughout a bulk sample^9^. Nevertheless, we are far from fully understanding the details of how atoms pack themselves densely with SRO, MRO, or even LRTO without translational periodic symmetry^10,11^, the degree of ordering in MGs can be intuitively and qualitatively estimated by the amplitude and the extension range of their oscillations in the radial distribution function obtained by diffraction techniques.

In practice, over the last few decades, intense efforts have been made to modulate the structures of MGs to obtain desired properties, primarily focusing on different synthesis routes or post-fabrication treatments. For instance, by carefully controlling the substrate temperature or deposition rate in magnetron-sputtering, ultrastable MGs were recently produced^12,13^, and a large elastic limit and enhanced glass temperature were realized^14^. Nano-glasses, synthesized by an inert gas condensation method, were found to be more ductile and catalytically more active^15,16^. Some MGs, after elastostatic loading, plastic deformation, ion irradiation, and cryogenic thermal cycling, have also shown improved plasticity^17^. Although the properties of such MGs can be considerably changed, the variations in their atomic structures are usually subtle and mainly limited to the SRO range, which is difficult to decipher with the traditional diffraction or imaging techniques. Moreover, pressure-induced first-order polyamorphic transitions have recently been extensively observed in MGs. These transitions mainly involve bond length or bond nature change in the SRO caused by electronic transition, but still, no obvious structural variances (in terms of ordering) are detectable over an extended atomic range^9,18–26^. In principle, the structural configuration space of a multicomponent MG is nearly infinite. Unfortunately, so far, the accessible states in the configuration space seem to be still extremely limited in MGs. Therefore, an intriguing question is raised: is it possible to effectively control and manipulate the structural order of MGs in the range of MRO and even beyond, i.e., freely mapping the configurational space?

In this work, using in situ high-energy XRD and small-angle x-ray scattering (SAXS) techniques, we study the high-temperature behavior of a Ce_65_Al_10_Co_25_ MG and confirm that a notable ordering process from SRO extended to MRO occurs during heat treatment prior to its primary crystallization. Highly ordered states in the Ce_65_Al_10_Co_25_ MG can be readily produced. Furthermore, by employing in situ high-pressure XRD and SAXS at room temperature, we find that the thermal-driven highly ordered states can be fully rejuvenated by applying pressure. The structural states of all the samples after ordering or disordering are characterized by HRTEM, ED, XRD, and differential scanning calorimetry (DSC). The electrical conductivity is also investigated for both ordering and disordering processes to demonstrate corresponding tunable properties with the structural evolution. Similar structural tuning also can be achieved in other MGs with different compositions, such as the La_65_Al_10_Co_25_ MG and the Ti_20_Zr_20_Hf_20_Cu_20_Co_20_ MG. These structural evolutions during temperature and pressure treatments demonstrate an effective two-way tuning of the atomic structures in MGs.

## Results

### High-temperature-induced structural ordering

**Fig. 1:**
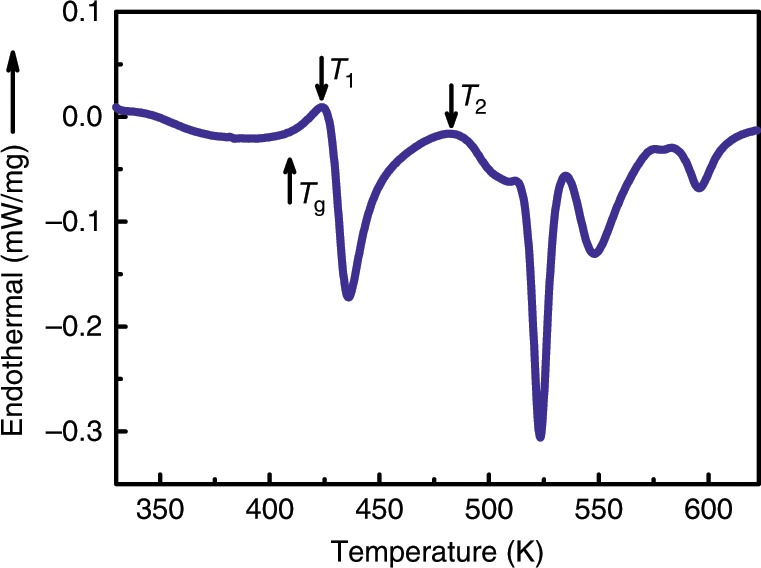
The DSC curve of the as-prepared Ce_65_Al_10_Co_25_ MG from 330 K to 625 K with a heating rate of 10 K per minute. The sample undergoes relaxation below the glass transition temperature, *T*_g_ ~ 410 K. The first exothermic peak starts at *T*_1_ ~ 428 K and is followed by three complex reactions starting at *T*_2_ ~ 483 K.

Figure 1 shows the DSC curve of the as-prepared Ce_65_Al_10_Co_25_ MG. The sample experienced obvious relaxation before the glass transition. The glass transition temperature, *T*_g_, ~ 410 K, is followed by four major exothermic peaks. The onset temperature of the first exothermic peak, *T*_1_, is ~ 428 K and ends at ~ 480 K, with an enthalpy release of ~ 9.7 J g^−1^. The subsequent three peaks, starting from *T*_2_, ~ 483 K, are not well separated and have a total enthalpy change of ~ 21.5 J g^−1^.

**Fig. 2:**
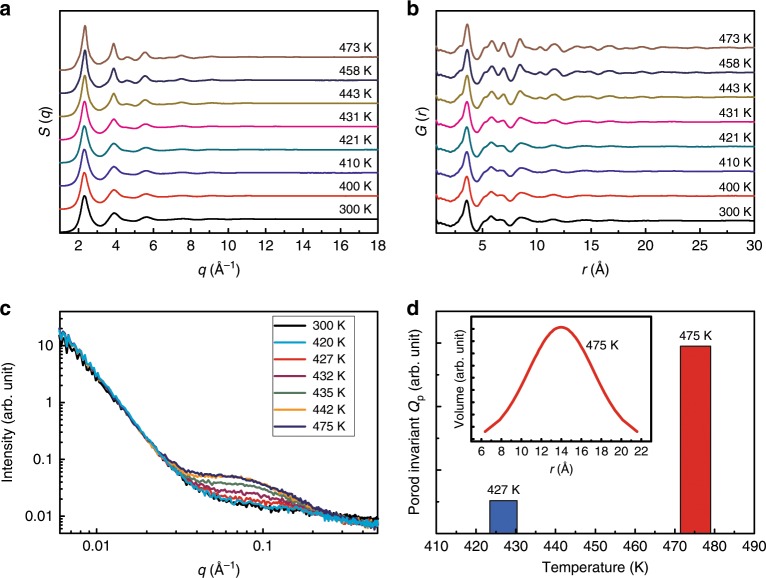
The structural evolution of the as-prepared Ce_65_Al_10_Co_25_ MG at high temperatures. **a** *S*(*q*) and **b** *G*(*r*) at representative temperatures. More features emerged in the patterns indicate that more ordered states were obtained during heating, but no sharp Bragg diffraction peaks were observed. **c** Representative SAXS data in the ordering temperature range, data at 300 K are also shown for comparison. The initial sample at 300 K is homogeneous according to its monotonically decayed data, whereas a hump at ~ 0.08 Å is observed when ordering starts above 427 K, implying density fluctuation emerges. **d** The comparison of Porod invariant at 427 K and 475 K relative to the value at room temperature, implying a considerable increasing of the volume of the heterogeneous scatterers upon heating from 427 K to 475 K. The size distributions of the scatterers at 475 K is shown in the inset, indicating an average radius limited to ~ 14 Å at this temperature.

To study the structural evolution during heating through these thermal events, in situ high-temperature high-energy XRD experiments were performed on the as-prepared Ce_65_Al_10_Co_25_ MG at beamline 11-ID-C at the Advanced Photon Source (APS), Argonne National Laboratory (ANL) (see Methods for details). Figure 2a shows the representative structure factor *S*(*q*) of the sample from room temperature to 473 K. No obvious change can be observed between 300 K and 421 K (below *T*_1_), which is expected as the relaxation and glass transition in MGs are usually not explicitly distinguishable by XRD^27^. Surprisingly, above *T*_1_, instead of developing to have sharp Bragg diffraction peaks, the *S*(*q*) only changes slightly in the peak width and intensity (Supplementary Fig. 1) with increasing temperature. This change is obviously differing from the typical crystallization process that occurs after entering the second exothermic event above *T*_2_ (Supplementary Fig. 2).

The Fourier-transformed reduced pair distribution functions, *G*(*r*), which provides the structural information in real space in terms of a probability distribution of interatomic pair correlations are shown in Fig. 2b. For the sample at 300 K, the oscillations in *G*(*r*) gradually vanish above ~ 15 Å, while *G*(*r*) above 421 K (~ *T*_1_) shows obviously enhanced oscillations at the medium range *r* space extended up to almost 25 Å. Obvious damping of oscillations in *G*(*r*) shown in all data suggests the absence of long-range translational symmetry. The narrowing and enhancement of the peaks in *S*(*q*) and the correspondingly more oscillations of *G*(*r*) in the larger but still limited *r* space with damping suggest another type of structural ordering rather than typical crystallization takes place right above 421 K. Thus, a highly ordered intermediate state before primary crystallization is observed in the Ce_65_Al_10_Co_25_ MG sample. Interestingly, this ordering process is irreversible, i.e., the highly ordered state can be readily retained to room temperature with a moderate cooling rate, implying that this highly ordered state might be in a solid state rather than another supercooled liquid.

To further understand the ordering process in this Ce_65_Al_10_Co_25_ MG, in situ high-temperature SAXS measurements were performed (see Methods for details). Figure 2c shows the SAXS data from 0.006 Å^−1^ to 0.5 Å^−1^ at representative temperatures. No obvious changes were found between RT and 420 K (~ *T*_1_). However, once above 420 K, a hump developed at ~ 0.08 Å^−1^, suggesting that density fluctuations occurred in the sample. Porod invariant ($Q_p = {\int} {I(q)q^2dq}$) is widely used to estimate the mass concentration directly from SAXS curves without any model assumption with respect to the particle shape^28^. Figure 2d shows the comparison of *Q*_p_ value at 427 K and 475 K relative to the value at room temperature, implying a considerable increase of the volume of the heterogeneous scatterers upon heating from 427 K to 475 K. By fitting the SAXS data using a spherical model, we were able to estimate the size distribution of the heterogeneous scatterers (a comparison of the fitting result with experimental data is shown in Supplementary Fig. 3). Even at the highest temperature of 475 K, the average radius of the highly ordered scatterers is limited to ~ 14 Å (inset of Fig. 2d). This implies that the ordering in the MG sample is associated with the formation of a highly ordered state with nano-scaled density fluctuation, which is a distinct phenomenon of medium-range ordering that involves the nucleation of a different state. It has been well recognized that the MGs have intrinsic spatial structural heterogeneity at quite a broad length scale from 2.5 nm to ~ 20 nm^29–32^. And it is suggested that the inhomogeneity in MGs may initialize the nucleation of shear bands during deformation^33^; therefore, it may provide nucleation sites for the ordering transition as we observed in this study.

### High-pressure-induced structural disordering

**Fig. 3:**
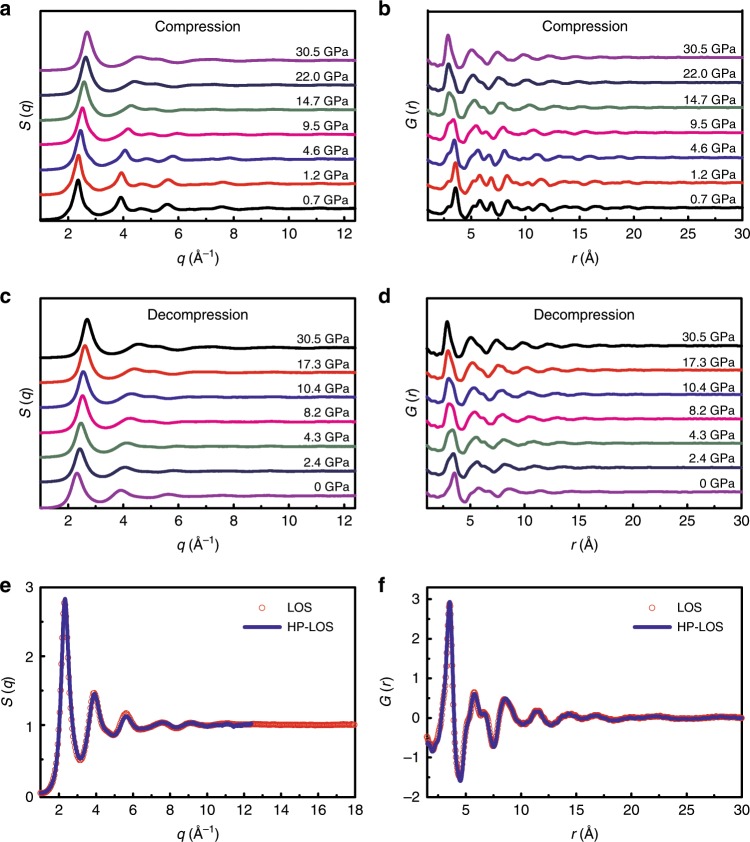
The structural evolution of the Ce_65_Al_10_Co_25_ HOS sample under high-pressure. **a** *S*(*q*) and **b** *G*(*r*) of the HOS sample during compression, **c** *S*(*q*), and **d** *G*(*r*) of the HOS sample during decompression. Fewer features in the *S*(*q*) and *G*(*r*) after high-pressure treatment indicate that thermal-driven high orderings are gradually erased by pressure. A comparison of the structures of the as-prepared sample (LOS) and the sample recovered from high-pressure experiments (HP-LOS) is shown in **e** *S*(*q*) and **f** *G*(*r*). The HP-LOS sample (blue line) almost coincides with the as-prepared LOS MG (red circle), suggesting highly similar structures.

Highly ordered Ce_65_Al_10_Co_25_ samples were obtained by heating to 458 K (< *T*_2_) at a rate of 10 K per minute, followed by cooling at a rate of ~ 50 K per minute to room temperature. Hereafter, the highly ordered sample recovered from 458 K annealing is referred to as a high-ordering state (HOS), and the initial as-prepared sample as a low-ordering state (LOS) for convenience. In situ high-pressure XRD experiments were performed on the HOS sample at room temperature using a symmetric diamond anvil cell (DAC) at beamline 13-ID-D at the APS, ANL (see Methods for details). Figure 3a–d show the *S*(*q*) and *G*(*r*) curves of the HOS sample at representative pressures during compression and decompression (a complete data set of the original in situ high-pressure XRD patterns is provided in Supplementary Fig. 4). When the pressure increased, the peaks in *S*(*q*) shifted to a higher *q* range owing to the densification effect (Fig. 3a). Meanwhile, more pronounced changes include the peaks at ~ 3.9, 4.7, 5.6, and 7.5 Å^−1^ either became much broader and weaker or almost disappeared. Correspondingly, the *G*(*r*) possesses fewer features and stronger damping of oscillations with increasing pressure (Fig. 3b). These changes suggest that the sample gradually becomes more disordered again during compression, which is consistent with the results of the in situ high-pressure SAXS experiment on the HOS sample, as shown in Supplementary Fig. 5, in which the hump as a characteristic feature of the highly ordered state weakened and eventually vanished when compressed up to 40.0 GPa.

During decompression, all the peaks remained broad, and the features in *S*(*q*) associated with the high-level ordering in the starting HOS sample did not reappear (Fig. 3c); also, strong oscillations did not show up again in the medium range of the *r* space (Fig. 3d). The thermal-driven ordering in the HOS sample is obliterated by an inverse high-pressure-induced disordering process. More interestingly, both *S*(*q*) and *G*(*r*) of the fully recovered sample from high-pressure coincide with those of the as-prepared LOS sample (Fig. 3e, f). This result demonstrates that after high-pressure treatment, the HOS sample can be basically converted back to the initial LOS irreversibly. Hereafter, we use HP-LOS to refer to the sample recovered from the high-pressure treatment of HOS. As long-distance atomic diffusion is hardly involved in the hydrostatic compression at room temperature (far below the glass transition temperature), the high similarity between the LOS and HP-LOS structures, strongly suggests that the thermal-driven LOS to HOS transition should be a chemically partitionless process. In addition, the HRTEM and selected area electron diffraction results (see Methods for details) rule out detectable regular crystalline lattice with long-range translational symmetry in all of the LOS and HOS (Supplementary Fig. 6).

### Property tuning accompanying structural evolution

**Fig. 4:**
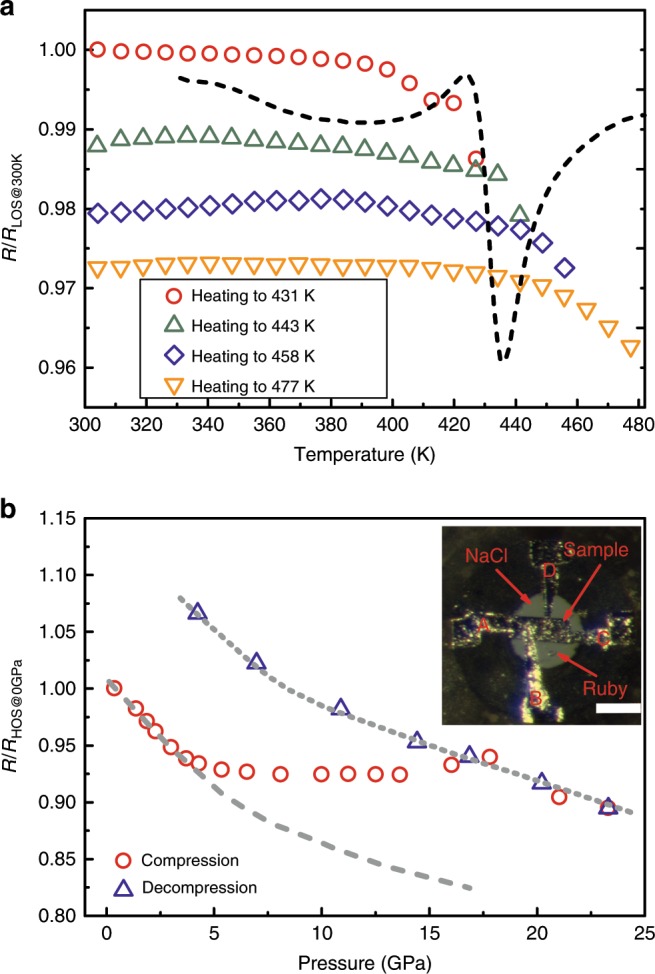
The resistance measurements of the Ce_65_Al_10_Co_25_ MGs during structural ordering and disordering. **a** The relative resistance changes of the Ce_65_Al_10_Co_25_ LOS sample during repeated heating to the different temperatures in the ordering peak of the DSC curve (shown with dash line). It is clear that the resistance decreases obviously once the sample was heated above *T*_1_, and it is irreversible and quenchable to room temperature. **b** The relative resistance change of the Ce_65_Al_10_Co_25_ HOS sample under compression and decompression. Two different states, HOS and LOS/HP-LOS, are indicated by dashed and dotted lines. The resistance of the sample firstly changes along the dashed line during compression, above ~ 5 GPa it deviates from the initial trend and eventually follows the new trend of the dotted line with a transition zone from ~ 5 GPa to ~18 GPa. The inset displays the image of the HOS sample in the DAC for resistance measurement together with four Pt foil electrodes and a ruby ball for pressure calibration. The scale bar represents 100 μm. The error bars for the resistance and pressure measurements are both smaller than the symbol size.

To demonstrate the capability of property tuning as structure changes, we measured the electrical transport property (resistance) change during ordering and disordering (see Methods for details), which is usually sensitive to structural order and relatively easy for in situ measurements. When heating up the initial LOS sample (as shown in Fig. 4a), below *T*_1_, the resistance only changes slightly. Once the temperature is above *T*_1_, dramatic resistance decreasing takes place continuously, the higher the temperature goes, the lower the resistance of the sample will be. This resistance decreasing is also irreversible when the sample is cooled down. Figure 4b shows the results of the high-pressure resistance measurement of the Ce_65_Al_10_Co_25_ HOS sample. The resistance firstly decreases below 5 GPa, which is typical behavior for normal MGs during compression, but it almost remains constant during further compressing and even slightly increases above ~ 16 GPa. According to the results shown in Fig. 3, above 5 GPa the structure is getting more and more disordered again. Therefore, it is reasonable that the disordering effect will compensate for the tendency of normal resistance decreasing owing to densification and results in almost constant resistance. Above certain pressure (~ 16 GPa) the disordering effect takes over the dominant role and the resistance gradually turns to increase. When the disordering process completes, above ~ 18 GPa, the resistance decreases with increasing pressure again, suggesting the HP-LOS sample also behaves like a normal MG. During decompression, the resistance reversely follows the HP-LOS trend, increasing monotonically with decreasing pressure. Two distinct evolution trends are clearly observed for HOS and HP-LOS states, indicated by dashed and dotted lines in the Fig. 4b respectively, with a transition zone between ~ 5 and ~ 18 GPa during compression.

## Discussion

MGs are thermodynamically metastable and will naturally and rapidly crystallize into stable crystals with long-range translational symmetry when heated above their crystallization temperatures. In Fig. 1, the first exothermic peak in the DSC curve has an enthalpy release of ~ 9.7 J g^−1^, which is comparable with typical crystallizations in MGs. Surprisingly, our diagnostic probes, including the in situ heating synchrotron radiation XRD and ex situ HRTEM experiments, did not detect the formation of any crystalline lattice with clear long-range periodicity. It is worth to note that a new glass transition-like endothermic signal was observed in the regular DSC measurement of the HOS sample and a similar one in the reversible heat flow in the temperature modulated DSC measurement (TMDSC) (Supplementary Fig. 7a and 7b). In addition, isothermal calorimetry experiments at around *T*_2_ revealed exothermic peaks (Supplementary Fig. 8a), which indicates a nucleation process taking place at *T*_2_, and a first-order phase transition separates the HOS from the final crystalline state above *T*_2_. Therefore, these results rules out the possibility of a vast pre-nucleation nano-embryos of the final stable crystals forming in the first exothermal event below *T*_2_, based on the method of Chen and Spaepen^34^ (i.e., quick grain-growth/coarsening from existing nanocrystal nuclei usually occurs in nano- or micro-crystalline solids, which results in a monotonically decaying DSC profile during isothermal annealing rather than a well-defined exothermic peak). All the above results from XRD, HRTEM, DSC, TMDSC, and the isothermal calorimetry measurements strongly suggest that the HOS phase is different from traditional nano-crystalline phases, but retains most of the non-crystalline characteristics. The oscillations in G(*r*) gradually extend to almost 25 Å during heating above *T*_1_, which may be associated with relative strong hybrid bonding nature as proposed in many multicomponent MGs rather than pure random hard-sphere contact^35,36^. Therefore, it suggests that the bonding/cluster forming in HOS may be stronger than LOS. Unfortunately, the atomic arrangement details of the HOS could not be determined by any of our accessible techniques at present.

Temperature-induced liquid-to-liquid polyamorphic transitions have been experimentally observed in some MG-forming liquids, suggested by heat capacity changes and the subtle kinks of the peak positions of the principal diffraction peak (PDP) in *S*(*q*) or nuclear magnetic resonance spectra as a function of temperature^37–41^. These transitions are usually reversible. Distinct liquid states can barely be recovered to form polyamorphic glasses for comprehensive study or practical applications at ambient conditions. We explicitly reveal two distinct structural states obtained by a thermal-driven irreversible ordering transition in the supercooled liquid region through an exothermal reaction. Similar anomalous exothermal reactions in the supercooled liquid regions have been observed in several different MG systems and different interpretations were introduced to explain such anomalies, e.g., phase separation in Pd-Ni-P MG^42^, irreversibly formation of local crystal-like ordering in Zr_36_Ti_24_Be_40_ MG^43^, long-period structures in Fe_48_Cr_15_Mo_14_C_15_B_6_Tm_2_ MG^44^, and local chemical short-range re-ordering in Fe_68_Mo_4_Y_6_B_22_ MG^45^ etc. Recent in situ experiments on a Pd_41.25_Ni_41.25_P_17.5_ MG^46^ reported that the long-debated origin of the anomalous exothermal reaction is a polyamorphic transition between two supercooled liquids, rather than a phase separation or a crystallization. Instead, Long et al.^47^ reported a nucleation and growth mechanism of a highly ordered, non-crystalline metallic phase (possessing no long-range translational symmetry) from an Al-Fe-Si melt during rapid cooling, providing another route to amorphous states formation. Our SAXS data of the Ce_65_Al_10_Co_25_ MG shows a nano-scale heterogeneous phase emerged as the HOS sample, whereas other probing methods reject the possibility of traditional nano-crystallization. Moreover, isothermal annealing the LOS sample around *T*_1_ also revealed exothermal peaks (Supplementary Fig. 8b), suggesting the ordering transition is a first-order nucleation-like process as well^34^.

Moreover, the ordering transition occurs at ~ 428 K (*T*_1_), which is in the supercooled liquid region of the initial LOS sample but is lower than the glass transition (a new glass transition at ~ 435 K, see Supplementary Fig. 7) of the HOS sample. Therefore, the ordering transition should be a transition from a supercooled liquid to a most likely metastable solid state with lower energy state and slower dynamics, and this metastable HOS further transforms into the stable crystalline state when heating above *T*_2_, which could explain the irreversibility of the ordering transition and the accompanying heat release. Actually, our results are highly reminiscent of the reentrant glass transition phenomena. Reentrant glass transitions are predicted by mode-coupling theory and have been extensively observed in colloid glass systems, where a repulsive glass changes into an attractive one with enhanced bonding and cluster formation, mediated by an intermediate liquid in between two glass states (a reduction in volume is equivalent to lowering temperature in colloidal systems)^48–50^. Therefore, a supercooled liquid to solid transition during heating does not necessarily mean crystallization as typically expected, which could result from a reentrant glass transition into another metastable glass state as well. The metastable states (HOS vs. LOS) dwell in different megabasins of the potential landscape. The transition between the two, upon limited (to avoid crystallization) thermal excitation, is not reversible in our case. However, pressure can be applied to alter the underlying potential landscape and tune the two distinct states. Although the experimental results including structural, calorimetric in this work point toward a highly ordered non-crystalline-like state, detailed structural information remains unknown. Therefore, development (or considerably increased volume fraction) of local crystal-like ordering clusters^51^ but still without translational symmetry in the HOS could not be fully eliminated.

In contrast to the natural tendency of MGs to lower their energy via relaxation or even crystallization, the cross-over from a relatively low-energy state (relaxed/aged) to a high-energy state (less relaxed/as-prepared), is also known as rejuvenation phenomena^52^, which can be realized by shot-peening^53^, irradiation^54^, severe plastic deformation^55^, creep^56^, high-pressure^57^, and axial compression^58^, etc. Ketov et al.^59^ discovered an interesting rejuvenation phenomenon in some MGs as a result of cryogenic thermal cycling treatment and believed the non-affine strain induced by thermal cycling was the underlying mechanism. In our work, the high orderings in the Ce_65_Al_10_Co_25_ HOS sample can be gradually but totally erased by a pressure-induced disordering transition with increased energy state (Fig. 3 and Supplementary Fig. 5). The high-pressure on the pressure-sensitive systems such as soft Ce-baring MGs would readily (otherwise may require higher pressures) generate/induce a much larger non-affine strain compared with the thermal cycling. Therefore, rejuvenation could take place in a way with much more dramatic structural tuning, as observed in this work.

Given the considerable extent of structural change during the two-way structural tuning, it is reasonable to expect that the properties of the MGs can be modulated accordingly. Although the electrical property of MGs is affected by many factors, in general, more ordered the system is, smaller the resistivity will be. The decreasing resistance in Fig. 4a is consistent with the irreversible structural ordering transition shown in Fig. 2. On the other hand, when the structure becomes more disordered, the resistivity will increase accordingly. These pressure-induced changes in resistance under high-pressure also agree well with the structure disordering shown in Fig. 3 and Supplementary Fig. 4. Therefore, these electrical resistance results demonstrate that two-way tuning of properties can be achieved as expected. It should be noted that the HP-LOS has relatively higher resistivity than the LOS if we use the HOS at ambient pressure as a reference. Almost identical *S*(*q*) and *G*(*r*) of LOS and HP-LOS do not necessarily guarantee exactly the same amorphous structure between them because the *S*(*q*) and *G*(*r*) only provide one-dimensional averaged structure information. The high-pressure treated HP-LOS sample may be within the same energy megabasin with LOS, but HP-LOS may store some extra elastic energy inside its structure after compression and decompression. More tunable properties are promising and worth extensive exploration in future work.

**Fig. 5:**
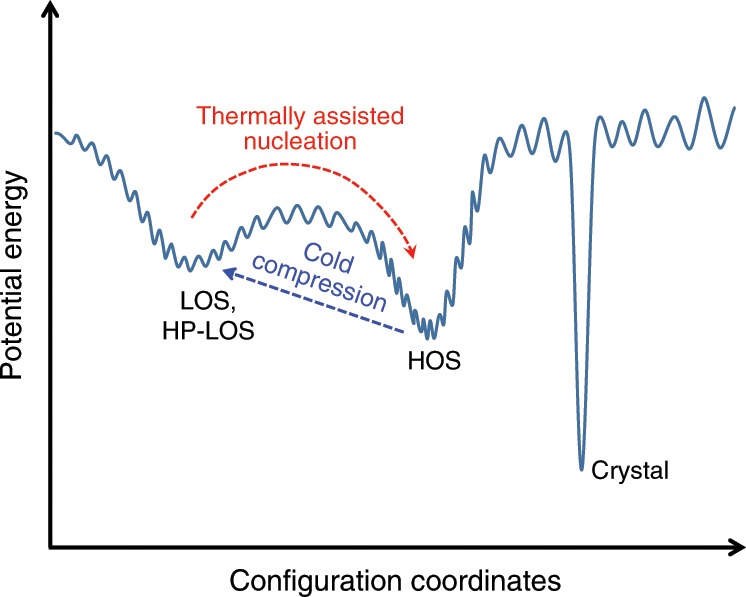
Schematic diagram of the potential energy landscape. The *x* axis represents all configuration coordinates. The three states labeled LOS/HP-LOS, HOS, and crystal differ in the shape and depth of their potential energy wells/megabasins. The thermal-driven transition from LOS to HOS and the pressure-driven transition from HOS back to LOS (HP-LOS) are indicated by the red and blue dashed arrows, respectively. Please note that the real PEL may be very complex and the transition path between different states is not necessarily limited in the plane of the paper.

To understand the two-way tuning of the structures in the Ce_65_Al_10_Co_25_ MG, we employed the schematic potential energy landscape (PEL) diagram^60^. Figure 5 illustrates the potential energy minima/megabasins and barriers between different configurations. Stable crystalline phases typically have the deepest energy minima and narrow well widths on the PEL. These deep and narrow energy minima will be easily bypassed during the rapid quenching of melt due to entropy barriers. Alternatively, the system will be trapped in many relatively high-energy metastable states, e.g., the LOS for Ce_65_Al_10_Co_25_ MG during melt quenching. The HOS state has more ordered structures, residing in another megabasin on the PEL with lower potential energy. Upon heating to *T* > *T*_1_, a first-order transition occurs via a thermally assisted nucleation process accompanied by energy release. As the HP-LOS is essentially indistinguishable in atomic structure from the LOS (probably within the same energy megabasin), the HOS to HP-LOS transition with energy restoration induced by cold compression can be regarded as the inverse process of the LOS to HOS transition. Likewise, Angell^61^ proposed similar transitions according to the experimental results in oxide network glasses, i.e., a transition from a high-energy state to a low-energy state in glasses may occur via a thermally assisted nucleation process, and an inverse transition could also take place during cold compression. Here, the two-way structural tuning of distinct states in the PEL proposed by Angell is realized in one multicomponent metallic system as well, providing a promising approach for studying the structural order and also tailoring desirable properties of MGs.

Our work reveals two-way structural tuning between two distinct states with different degrees of ordering in an MG. This two-way tuning of MGs is achieved via first-order transitions by altering temperature and pressure, which differs itself from the traditional aging/rejuvenation phenomena with subtle structural modification most likely limited in the same energy megabasin. Also, two-way tuning of MG structures is by no means limited to this specific composition; it could be generally used in many MG systems with anomalous exothermal reactions in supercooled liquid regions and a big difference in the bulk moduli of constituent elements. For instance, by simply substituting Ce element with La (no 4*f* electron), highly similar phenomena can be observed in the La_65_Al_10_Co_25_ MG (Supplementary Fig. 9). Another example of such an ordering-disordering two-way tuning phenomenon is also observed in a Ti_20_Zr_20_Hf_20_Cu_20_Co_20_ MG (Supplementary Fig. 10). Therefore, by combining temperature and pressure, we demonstrate that it is possible to tune the structures (ordering/disordering) in MGs to a great extent into many other metastable states that have never been achieved or explored before, which may also lead to the discovery of various MG structures (states) that are inaccessible by traditional routes. Moreover, finding this method of sampling the PEL of a multicomponent metallic system may also shed light on the nature of glass formation, the structural–properties relationship in MGs or even glasses in general.

## Methods

### Sample preparation and DSC measurement

The master alloy of a nominal composition of Ce_65_Al_10_Co_25_ (at.%) was prepared by arc-melting using high purity raw metals under a Ti-gettered argon atmosphere. The Ce_65_Al_10_Co_25_ MG ribbon samples with a thickness of ~ 30 μm were prepared by single-roller melt spinning. The thermally annealed HOS samples used in the in situ high-pressure experiments were prepared by heating the as-prepared LOS ribbon samples to 458 K in a Linkam THMS600 heating stage with flowing ultrahigh purity Ar as the protecting gas at a heating rate of 10 K per minute and then cooling down to room temperature at a cooling rate of 50 K per minute. The thermal analysis was conducted using a DSC Perkin-Elmer DSC 8000 with a heating rate of 10 K per minute. The reversible and irreversible components of the total heat flow of Ce_65_Al_10_Co_25_ HOS sample were determined in a Perkin-Elmer 8500 DSC using a Stepscan program from 325 K to 625 K. Temperature was increased with a heating rate of 5 K per minute and held isothermally at every 1 K interval for 60 s.

### In situ high-temperature high-energy XRD

In situ high-temperature high-energy XRD experiments were performed at beamline 11-ID-C at the APS, ANL with an x-ray wavelength of 0.1174 Å and a beam size of 0.5 × 0.5 mm^2^. The as-prepared Ce_65_Al_10_Co_25_ MG was heated at a rate of 10 K per minute in a Linkam THMS600 heating stage with flowing ultrahigh purity Ar as the protecting gas. The exposure time was set to 15 s, and the temperature variation with each XRD pattern was around 2.5 K. The two-dimensional (2D) diffraction images were collected continuously during heating by a Perkin-Elmer amorphous silicon detector, with a maximum wave vector momentum transfer of *q* = 18 Å^−1^. Then, the 2D images were integrated with the software Fit2D^62^ and Dioptas^63^, and the structure factor *S*(*q*) and reduced pair distribution function *G*(*r*) were obtained using the program package PDFgetX3^64^.

### In situ high-temperature SAXS

In situ high-temperature SAXS experiments were performed at beamline 12-ID-B at APS, ANL with x-ray energy of 13.3 keV. The as-prepared Ce_65_Al_10_Co_25_ MG with a thickness of ~ 18 μm was heated at a rate of 10 K per minute in a Linkam THMS600 heating stage with flowing ultrahigh purity Ar as the protecting gas. The exposure time was set to 2 s for each pattern. The 2D scattering images were collected continuously by a Pilatus 2 M detector during heating. By removing the samples, background scattering patterns of the heating stage were also collected before and after the experiment. The data were integrated by a built-in code in Matlab first and then analyzed using the Irena package^65^. Since no clear evidence of the specific shapes of the heterogeneous scatterers was achieved, a spherical model was adopted for simplicity.

### In situ high-pressure XRD

The HOS sample was cut into small pieces of ~ 25 × 25 μm^2^ and loaded into a symmetric DAC to perform high-pressure experiments. Helium was loaded as the pressure transmitting medium and small ruby balls were also loaded near the samples as the pressure calibrant. The in situ high-pressure XRD experiments were carried out at beamline 13-ID-D at APS, ANL. A monochromatic x-ray beam with a wavelength of 0.3100 Å was focused down to ~ 3.5 × 4 μm^2^ by a Kirkpatrick-Baez (KB) mirror system. 2D diffraction images were collected with a Mar165 charge-coupled device (CCD) detector. To obtain the largest possible *q* range, the CCD was shifted to one side and the symmetric DAC was rotated accordingly to reach the largest coverage of the two-theta angles. A maximum *q* of 12.4 Å^−1^ was obtained in the high-pressure XRD experiments. Although the two-theta coverage is still not as large as the in situ high-temperature XRD experiment, it is evidenced to be large enough for MGs to extract reliable *S*(*q*) and *G*(*r*) in high-pressure experiments^66,67^. The exposure time for each pressure point was 30 s, and 900 s for the PDF measurements at selected pressure points to get better statistics. The background scattering from the high-pressure environment was also taken next to the sample, where the x-ray beam only went through the pressure medium helium and two diamonds anvils. The in situ ultrahigh-pressure XRD experiment of the annealed Ti_20_Zr_20_Hf_20_Cu_20_Co_20_ MG was also carried out at beamline 13-ID-D with diamond culets of 200 μm. No pressure medium was used in this experiment and a gold foil was used to calibrate the pressure. In situ high-pressure XRD of the La_65_Al_10_Co_25_ MG in the supplementary material was performed in the beamline 12.2.2 at Advanced Light Source (ALS) in Lawrence Berkeley National Laboratory (LBNL) with the x-ray wavelength of 0.4959 Å and the beam size of 10 × 10 μm^2^. The 2D XRD images were processed using the same software packages mentioned in the in situ high-temperature XRD experiments.

### In situ high-pressure SAXS

In situ high-pressure SAXS experiments were performed at beamline 12-ID-B at APS, ANL with x-ray energy of 14.0 keV. The beam size was focused to ~ 180 × 30 μm^2^. The HOS sample with a thickness of ~ 15 μm was cut into small pieces of ~ 150 × 50 μm^2^ and loaded into a symmetric DAC to perform high-pressure experiments. A 4:1 (volume ratio) methanol–ethanol mixture was loaded as the pressure transmitting medium. A Re gasket with a 190 μm diameter hole was used. Both the sample and ruby balls were loaded on the lower side of the hole and the upper side was left for background collection (the normal direction of the hole is parallel to the beam path). At each pressure, SAXS patterns were collected with an exposure time of 2 s, and accumulated five times for better statistics. The 1D profiles were obtained using the same software packages mentioned in the in situ high-temperature SAXS experiments.

### HRTEM

To avoid any high-energy process induced artifacts in typical HRTEM amorphous sample preparation, we carefully prepared our samples by mechanically comminuting the MG samples in liquid nitrogen, and then dispersed these crushed tiny flakes in ethyl alcohol and picked them up using a holey carbon grid. The prepared HRTEM sample was quickly transferred into the TEM to minimize possible oxidation. HRTEM experiments were carried out using the Chromatic-Aberration Corrected TEM (ACAT) at ANL with an image corrector to correct both spherical and chromatic aberrations. An accelerating voltage of 80 kV was used to minimize the possible electron irradiation effect on the sample structure.

### In situ high-temperature/pressure resistance measurement

The standard four-probe method was used for both high-temperature and high-pressure resistance measurements using an AC current source and a nano-voltmeter. The high-temperature resistance was performed in a Linkam HFS600E-PB4 probe stage. To ensure the shape of the ribbon sample remains unchanged during heating and cooling, the sample was securely sandwiched between two thin glasses. To check if the resistance change is also irreversible as the structural ordering transition. The sample was repeated heating and cooling to targeted temperatures in the ordering peak (defined in the DSC curve) with a heating rate of 20 K per minute and a cooling rate of 150 K per minute following the temperature sequences of 300 K–431 K–300 K–443 K–300 K–458 K–300 K–477 K. High-pressure resistance measurements were performed in a DAC with anvil culet size of ~ 400 μm. A T301 stainless steel gasket was pre-indented to ~ 20 GPa, and a hole of ~ 360 μm was drilled inside the indent. Then, the hole was filled with a mixture of cubic-BN (cBN) and epoxy that was compressed to ~ 25 GPa, reliably insulating the sample and electrodes against the gasket. A hole of 150 μm was drilled again in the indent of the cBN + epoxy and then filled with NaCl used as a pressure transmitting medium. A Ce_65_Al_10_Co_25_ HOS ribbon sample was cut to the size of ~ 130 × 50 μm^2^ and placed on the NaCl. Four electrodes made of Pt thin foil (~ 4 μm in thickness) (A, B, C, and D in the inset of Fig. 4b) were implanted for the current source and voltage measurement.

## Supplementary information

Supplementary Information

## Data Availability

The data that support the findings of this study are available from the corresponding authors upon request.
