# Supplementary material for: Two-way tuning of structural order in metallic glasses
Source: Nat Commun. 2020 Jan 16;11:314. doi: 10.1038/s41467-019-14129-7 (PMC6965127; doi:10.1038/s41467-019-14129-7)
Supplement: Supplementary file 1 — Supplementary Information [file 41467_2019_14129_MOESM1_ESM.pdf]

Supplementary information for

Two-way tuning of structural order in metallic glasses

Lou *et al*.

This file includes supplementary Figs. 1-10.

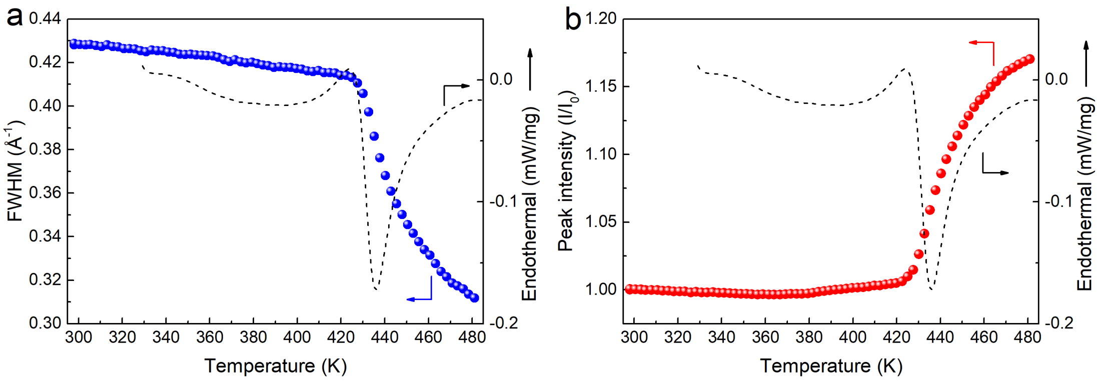

Supplementary Figure 1. The evolution of the principal diffraction peak (PDP) in *S*(*q*) as a function of temperature compared with the DSC curve. (a) The full width at the half maximum (FWHM) and (b) the normalized peak intensity. The principle peaks were fitted by a Voigt line profile. The FWHM only decreases slightly during heating to T_1 as relaxation occurs (a decrease of ~2.3%). In contrast, above T_1, the FWHM decreases quickly from ~0.43 Å^{-1} at 421 K to ~0.33 Å^{-1} at 473 K (a decrease of 23%). Meanwhile, the peak intensity also begins to significantly increase above 421 K (a quick increase of ~17%). The dramatic changes of the peak width and peak intensity above 421 K are closely correlated with the first exothermic event starting at T_1 ~428 K, shown in the DSC curve. Similar substantial changes in the peak width and intensity of *S*(*q*) were also observed in Ref. 13 between ordinary and ultrastable glasses.

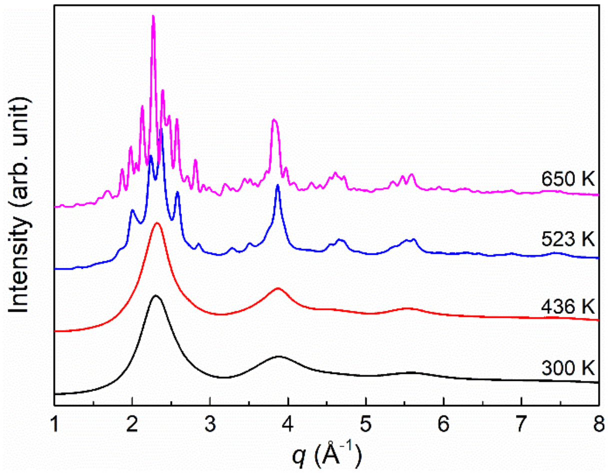

Supplementary Figure 2. A comparison of four representative XRD patterns of the $Ce_{65}Al_{10}Co_{25}$ MG samples during heating up to 650 K. The XRD pattern of 436 K (at the center of the first exothermal peak in Fig. 1) is only slightly different from the pattern at 300 K (the initial as-prepared glass state). However, it is totally different from the characteristic XRD patterns of the crystallized samples with plenty of sharp Bragg diffraction peaks as a result of lattice periodicity.

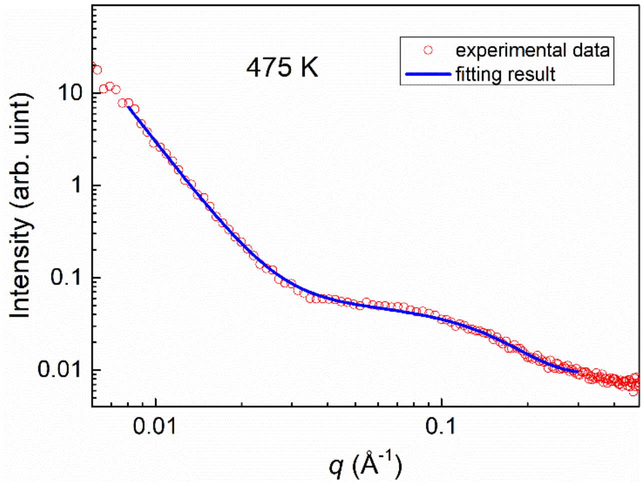

Supplementary Figure 3. Comparison of experimental and the spherical model fitted curves of the SAXS data at 475 K.

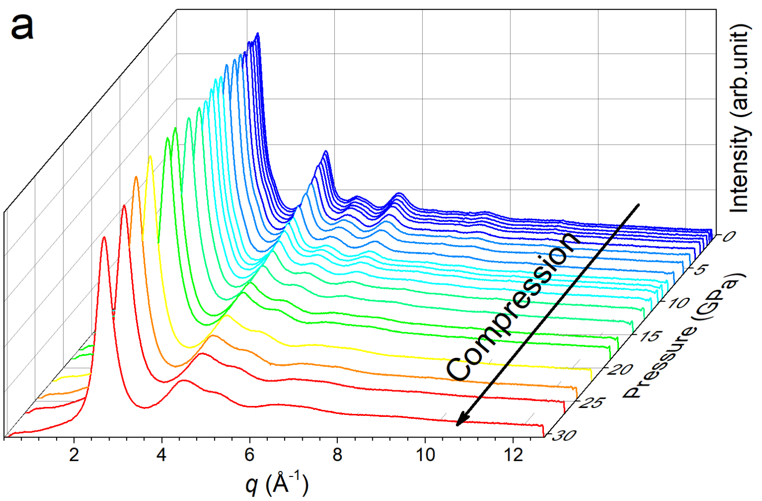

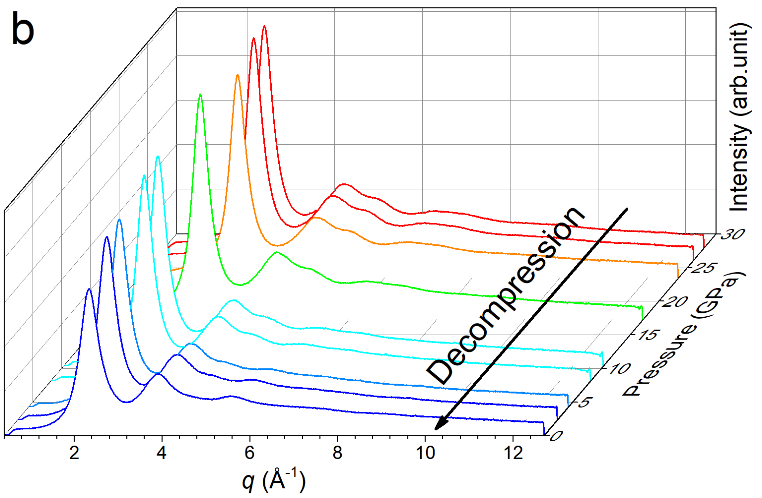

Supplementary Figure 4. The XRD patterns of the HOS sample as a function of pressure during (a) compression and (b) decompression.

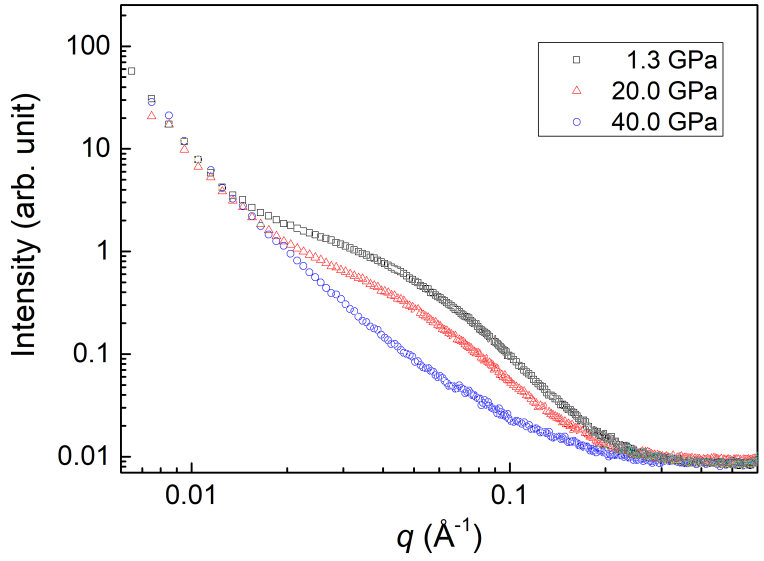

Supplementary Figure 5. SAXS data of the HOS sample under high-pressure. The hump corresponding to the highly ordered phase in the HOS sample is suppressed when compressed from 1.3 GPa to 20 GPa. The hump totally disappears at 40 GPa, and the data monotonically decays like the data of the LOS sample at 300 K, shown in Fig. 2c.

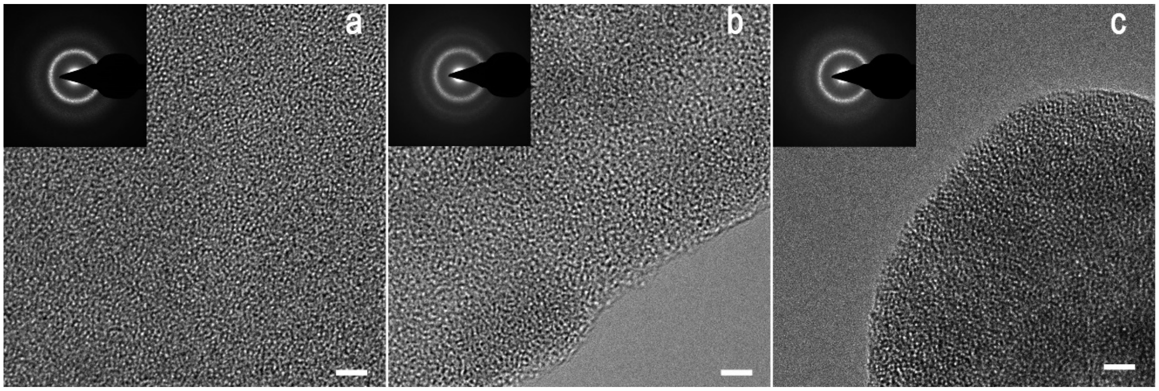

Supplementary Figure 6. HRTEM images of the $Ce_{65}Al_{10}Co_{25}$ MG sample at different states. HRTEM images of $Ce_{65}Al_{10}Co_{25}$ MGs in the (a) as-prepared (LOS), (b) 458 K heated (HOS), and (c) high-pressure recovered (HP-LOS) states. The scale bars in each image represent 2 nm. The insets show the corresponding SAED patterns. All the HRTEM images of the three samples show characteristic random patterns of amorphous materials without any identifiable symmetry or sharp contrast, which does not support the possibility of typical nano-crystal formation in the HOS sample. The selected area electron diffraction (SAED) patterns of the LOS and HP-LOS are very similar, while the SAED of the HOS exhibits a relatively stronger second peak than the LOS and HP-LOS, which is consistent with the results of the XRD experiments.

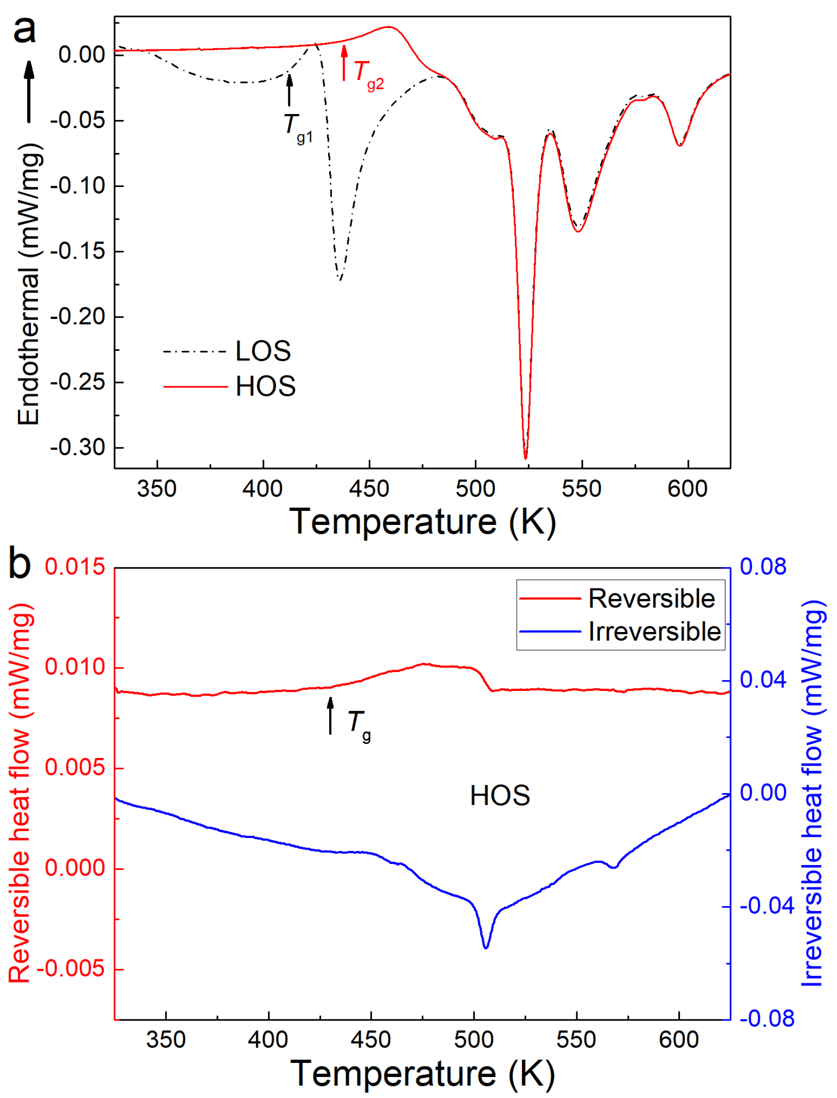

Supplementary Figure 7. DSC curves of the $Ce_{65}Al_{10}Co_{25}$ HOS sample. (a) Comparison of the DSC curves of the as-prepared LOS sample and HOS sample recovered from 458 K. The two DSC curves coincide above 486 K, where the first exothermic event ends. Below 486 K, the two samples behave totally differently, the HOS sample shows no relaxation before its glass transition but only an enhanced glass-transition-like signal at ~ 435 K. (b) Reversible and irreversible heat flow of the HOS sample in the temperature modulated DSC measurement. Again, a glass-transition-like signal is clearly observed at ~ 430 K in the reversible heat flow curve which gives evidence that the HOS sample has typical glass characteristics. The small crystallization peaks merged together in the irreversible curve due to the low overall heating rate.

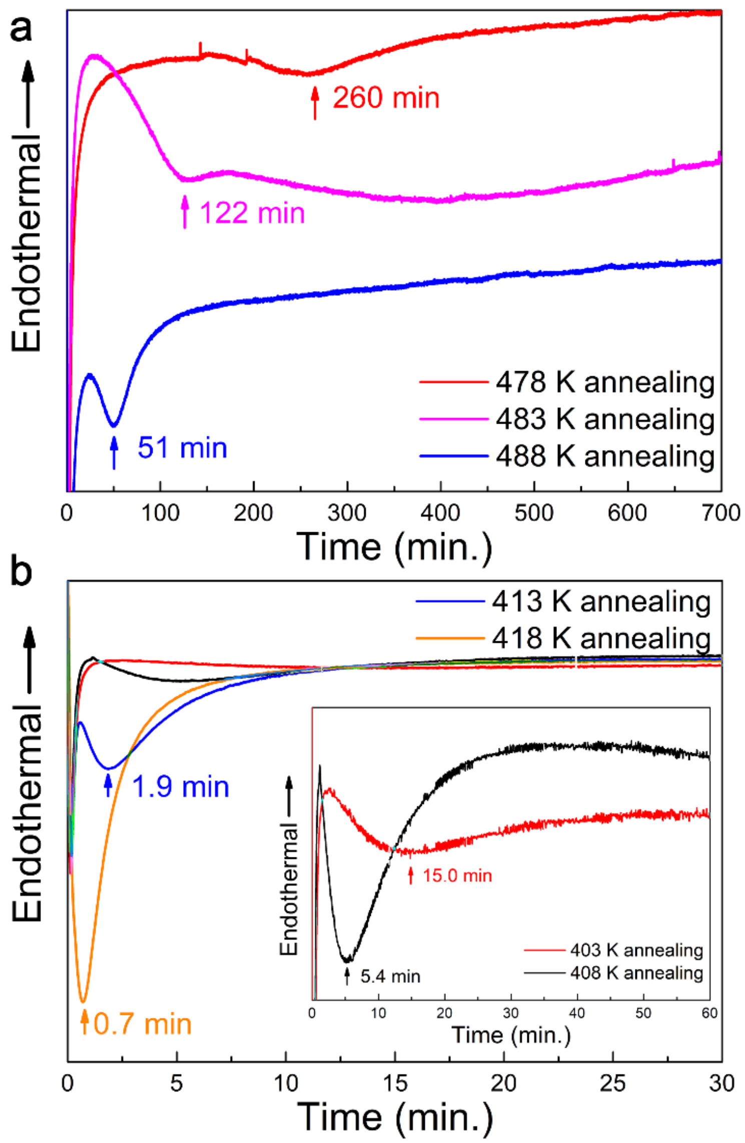

Supplementary Figure 8. The DSC curves of (a) the HOS sample during isothermal annealing around T_2, and (b) the as-prepared LOS sample during isothermal annealing around T_1 as a function of time. An exothermic peak occurs at the non-zero time in each DSC curve, demonstrating the nucleating nature of both the transitions corresponding to the first and second exothermic events in Fig.1.

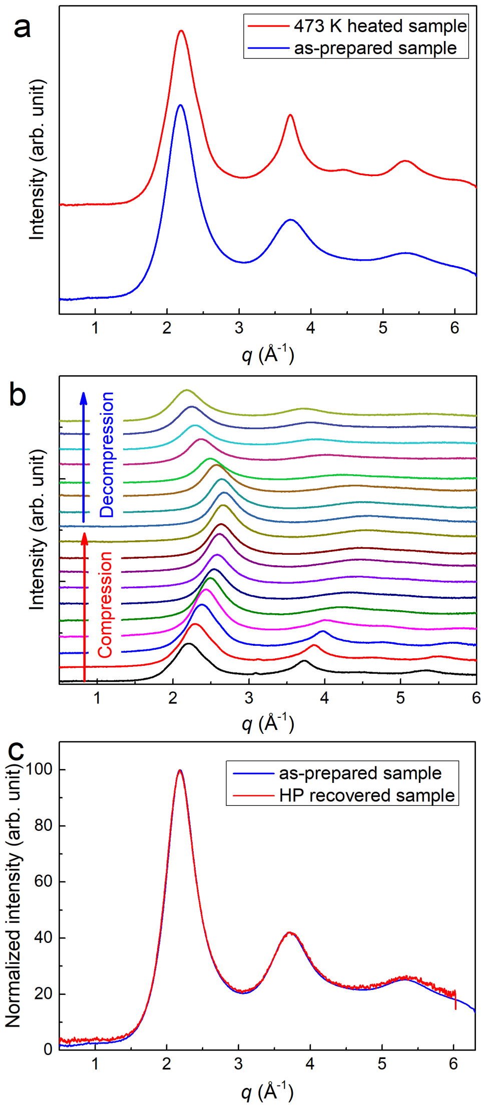

Supplementary Figure 9. The ordering and disordering transitions of the La65Al10Co25 MG. (a) Comparison of the XRD patterns of the as-prepared and heated (473 K) La65Al10Co25 MGs, showing the heated (473 K) sample is not crystallized but apparently more ordered than the as-prepared one. (b) The in situ high-pressure XRD patterns of the 473 K heated La65Al10Co25 MG up to 47.9 GPa. The features associated with high-orderings were gradually removed upon compression and did not reappear during decompression. (c) Comparison of normalized XRD patterns of the as-prepared sample and the high-pressure recovered sample, showing almost identical XRD structure, indicating the totally erasing of the high-orderings previously induced by heating. These results demonstrate that the two-way structural tuning in MGs is not limited to a specific composition and not necessary to involve cerium as a component in MGs, but can be extended to more other MG systems.

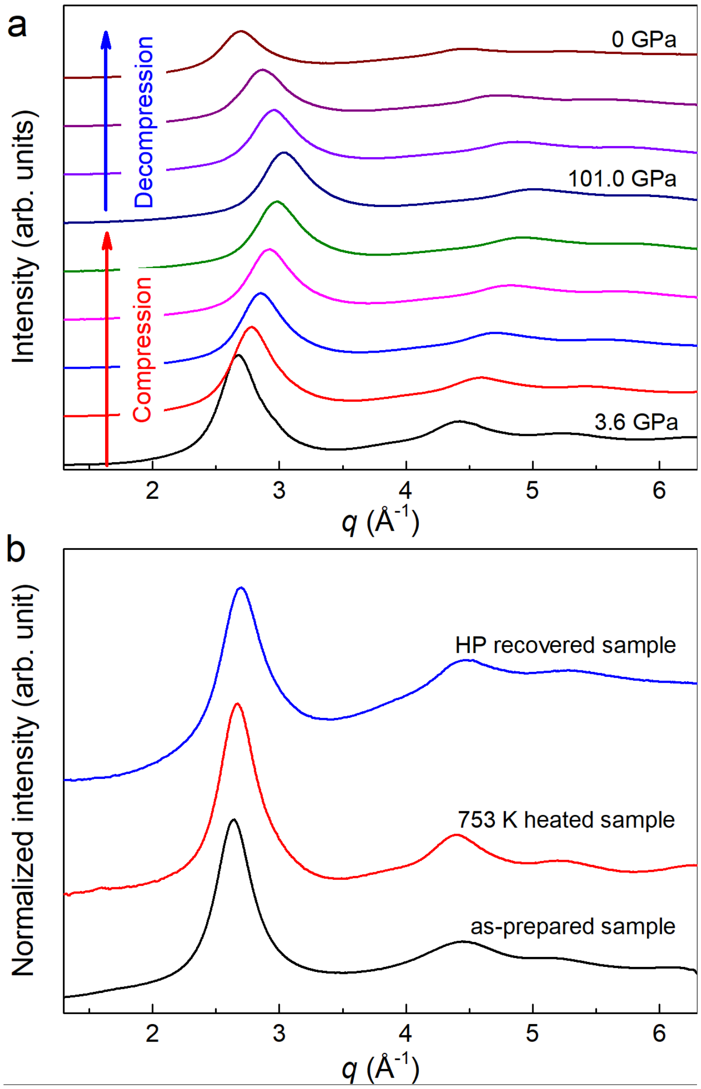

Supplementary Figure 10. The ordering and disordering transitions of the $Ti_{20}Zr_{20}Hf_{20}Cu_{20}Co_{20}$ high-entropy MG. (a) The *in situ* high-pressure XRD patterns of the 753 K heated $Ti_{20}Zr_{20}Hf_{20}Cu_{20}Co_{20}$ MG up to 101.0 GPa. The features associated with high-orderings introduced by heating were gradually removed upon compression and did not reappear during decompression. (b) Comparison of normalized XRD patterns the as-prepared sample, 753 K heated sample, and the high-pressure recovered sample. The 753 K heated one is not crystallized but is more ordered than the as-prepared one. After ultra-high pressure (101.0 GPa) treatment, the sample becomes more disordered. These results further suggest that the two-way structural tuning can be found in more MG systems other than rare-earth-based systems.
